# Lessons from Two Early COVID-19 Hospital Outbreaks in Germany to Inform Strategies for Controlling Emerging Nosocomial Outbreaks of Highly Transmissible Respiratory Viruses

**DOI:** 10.3390/jcm15062290

**Published:** 2026-03-17

**Authors:** Sofia Burdi, Felix Reichert, Barbara Mühlemann, Victor M. Corman, Terry C. Jones, Martin Hölzer, Susanne B. Schink, Patrick Larscheid, Jakob Schumacher, Gudrun Widders, Inas Abdelgawad, Christian Brandt, Nicole Dinsel, Katharina Jelavic, Nadine Kurzke, Jörg Hofmann, Janine Michel, Annika Brinkmann, Stephan Fuchs, Christian Drosten, Tim Eckmanns, Muna Abu Sin

**Affiliations:** 1 Department of Infectious Disease Epidemiology, Robert Koch Institute, 13353 Berlin, Germany; 2 Postgraduate Training for Applied Epidemiology (PAE), Department of Infectious Disease Epidemiology, Robert Koch Institute, 13353 Berlin, Germany; 3 European Programme for Intervention Epidemiology Training (EPIET), European Centre for Disease Prevention and Control (ECDC), 16973 Stockholm, Sweden; 4 National Consultant Laboratory for Coronaviruses, 10117 Berlin, Germany; 5 Institute of Virology, Charité Universitätsmedizin, 10117 Berlin, Germany; 6 German Centre for Infection Research (DZIF), Partner Site Charité, 10117 Berlin, Germany; 7 Genome Competence Center, Robert Koch Institute, 13353 Berlin, Germany; 8 Local Public Health Agency Reinickendorf, 13407 Berlin, Germany; 9 Local Public Health Agency Spandau, 13597 Berlin, Germany; 10 Forensic Medicine and Clinical Toxicology Department, Faculty of Medicine, Cairo University, Al Kasr Al Aini, Cairo 4240310, Egypt; 11 Public Health Authority Havelland, 14712 Brandenburg, Germany; 12 Institute for Hygiene and Environmental Medicine, Vivantes Hospital, 13407 Berlin, Germany; 13 Centre of Infectious Diseases, Medical Microbiology and Hygiene, Medical Faculty Heidelberg, 69120 Heidelberg, Germany; 14 Labor Berlin-Charité Vivantes, 13353 Berlin, Germany; 15 Center for Biological Threats and Special Pathogens 1 (ZBS 1), Robert Koch Institute, 13353 Berlin, Germany

**Keywords:** healthcare-associated infections, infectious disease outbreak, infection prevention and control, respiratory infections, phylogenetic analysis

## Abstract

**Background/Objectives**: Nosocomial outbreaks of viral respiratory infections strain healthcare systems and endanger patients and healthcare workers (HCWs). We describe two large nosocomial outbreaks with the SARS-CoV-2 Alpha variant, during its initial emergence in Germany, to assess transmission dynamics, effectiveness of control measures, and challenges in managing highly transmissible respiratory viruses. **Methods**: Confirmed cases were inpatients, HCWs, or their contacts testing SARS-CoV-2-positive since 1 January 2021 (Hospital A [HA])) or 21 January 2021 (Hospital B [HB])) with N501Y and delH69/V70 spike gene mutations. We conducted case interviews, reviewed medical records and shift schedules, and performed sequencing, genome reconstruction, and phylogenetic analysis. We describe cases, transmission chains, and control measures. **Results**: HA reported 18 patient cases, 20 HCW cases, and 33 community cases (N = 71). HB reported 48 patient cases, 43 HCW cases (13 in a COVID-19 ward), and 27 community cases (N = 118). In-hospital transmission occurred patient-to-patient, HCW-to-patient, patient-to-HCW, and HCW-to-HCW. HA halted admissions immediately after the initial cases; HB implemented measures gradually. Regular testing detected pre-symptomatic (HA = 6; HB = 18) and asymptomatic cases (HA = 3; HB = 13). Testing of agency staff was incomplete. The suspected primary case was an HCW in HA and a patient in HB who required resuscitation shortly after admission. **Conclusions**: Early COVID-19 outbreaks offer valuable lessons for managing emerging nosocomial outbreaks of highly transmissible respiratory viruses. Our findings provide empirical evidence for effective interventions, including rapid response, testing, HCW protection, and rigorous contact tracing in high-risk emergency situations. Managing agency staff remains a major challenge.

## 1. Introduction

Nosocomial respiratory virus outbreaks significantly impact patients, delay treatment, prolong hospital stays, increase morbidity and mortality, strain healthcare resources, and may contribute to community spread [1,2,3,4,5]. Reducing SARS-CoV-2 transmission in healthcare settings is challenging due to the virus’s high transmissibility, asymptomatic transmission, long incubation period, and viral evolution [6,7,8]. Patient–healthcare worker (HCW) interactions add risk, worsened during the pandemic by HCW overburdening and high community incidence rates reflected within hospitals [9,10].

The SARS-CoV-2 lineage Alpha (B.1.1.7) was first described in the United Kingdom, where it showed increasing spread since early December 2020 [11,12]. At the end of December 2020, the Alpha variant was reported for the first time in Germany, and, despite efforts to prevent further spread, it became the most prevalent strain at the beginning of March 2021 [13,14]. The details of its emergence have been thoroughly studied, showing that the highly transmissible Alpha variant led to a rapid increase in morbidity and mortality [15,16,17].

On 18 January 2021, three SARS-CoV-2 Alpha cases were detected in a tertiary hospital in Germany (Hospital A, HA). As more cases emerged, an outbreak team was formed on 22 January 2021, including hospital representatives, particularly the infection prevention and control (IPC) team, the responsible public health authority (PHA), laboratories and the national public health institute, to investigate and manage the outbreak. On 22 January 2021, a patient in a tertiary hospital (Hospital B, HB) in a neighboring district tested positive for the Alpha variant. As further cases emerged among the patients and staff on different wards of HB, another outbreak investigation team was formed.

Here, we describe the two nosocomial outbreaks caused by the emerging SARS-CoV-2 Alpha variant that occurred in January 2021 in two hospitals in the same city in Germany. Our objective is to describe the outbreaks and the measures taken to contain them and mitigate community spread, specifically addressing the challenges posed by a highly transmissible respiratory virus. This analysis aims to extract insights for refining infection control strategies for emerging respiratory virus outbreaks in acute-care settings.

## 2. Materials and Methods

### 2.1. Study Design

This study is a descriptive outbreak analysis of two nosocomial SARS-CoV-2 Alpha variant outbreaks that occurred in January–February 2021 in two tertiary hospitals in Germany.

### 2.2. Setting

HA and HB are both tertiary hospitals in neighboring districts of the same city in Germany. HA had 694 beds and 1574 permanent staff, employing 100 full-time agency staff. HB had 646 beds, 1417 permanent staff, and 163 full-time agency staff.

### 2.3. Case Definition

A confirmed case was defined as a patient or HCW who had been hospitalized or employed since 1 January 2021 in HA or since 21 January 2021 in HB, or a contact person of a patient or HCW as defined above, all with a sequence-confirmed Alpha variant or a PCR-positive variant with S:N501Y and S:delH69/V70 mutations in the spike (S) gene and symptom onset on or after 1 January 2021 or 21 January 2021, respectively (or date of sampling if asymptomatic or date of symptom onset not available).

A possible case was defined as a person with SARS-CoV-2 detection using PCR or rapid antigen test and disease onset (or date of sampling) on or after 1 January 2021 for HA or 21 January 2021 for HB without further typing or sequencing with a strong epidemiological link to a confirmed case. A strong epidemiological link is fulfilled if the person was a contact person to a confirmed case up to 14 days before the onset of the disease.

The start of the time criterion differed between hospitals due to differences in detection of first Alpha cases, variant screening implementation, and retrospective testing. In HA, routine mutation screening was introduced in calendar week 02/2021, and the first Alpha cases were detected on 18 January 2021. Previously stored positive samples dating back to 1 January 2021 were retrospectively re-tested. In HB systematic mutation screening was already established, and retrospective case finding was subsequently not conducted beyond 21 January 2021.

Contact persons were defined as individuals exposed to a case regardless of exposure duration, type, or face masks used. This definition included all contact categories at the time and reflected a precautionary approach during the early emergence of the Alpha variant to prevent further transmission, including spread into the community.

### 2.4. SARS-CoV-2 Specimen Testing and Phylogenetic Analysis

From calendar week 02/2021, PCR-positive specimens—and available retrospective specimens—were tested for S:delH69/V70 and S:N501Y mutations. All diagnostic samples that tested positive for the N501Y and/or delH69/V70 spike gene mutations were flagged for next-generation sequencing [18]. We attempted sequencing for all mutation-positive samples; sequences with >90% genome coverage were included in the analyses. Samples with a higher viral load were sequenced natively without specific PCR amplification. For samples with lower viral load, we employed a SARS-CoV-2-specific PCR amplicon-based sequencing approach using Nimagen (NimaGen B.V., Nijmegen, The Netherlands) or ARTIC protocols [19] and the primers and protocol by Brinkmann et. al. [20]. Sequencing was performed using Illumina (Illumina, Inc., San Diego, CA, USA) or Oxford Nanopore (Oxford Nanopore Technologies, Oxford, UK) sequencing platforms.

Reads were aligned to sequence EPI_ISL_402125 (GISAID accession no.) using bowtie2 with the –no-unal and –local arguments [21,22]. Consensus sequences were called using iVar (version 1.3.1) with the -m 5 and -t 0.6 options [23]. Sequences were aligned with MAFFT (version 7.471) using the --auto --addfragments option, with the sequence EPI_ISL_402125 as the reference [24]. Maximum likelihood phylogenetic trees were inferred using IQ-TREE (version 2.2.0.3) [25]. The substitution model was determined by performing an initial IQ-TREE run on the full alignments with the -m MF -nt AUTO options. This determined the K2P substitution model as optimal for the alignments. Subsequent IQ-TREE runs were performed using the -m K2P -bb 1000 -alrt 1000 -nt AUTO options, performing 1000 ultrafast bootstraps.

### 2.5. Epidemiological Investigations and Transmission Network

In the descriptive epidemiological analyses, including case characteristics, secondary attack rates (SARs), and transmission network reconstruction, both confirmed and possible cases were included.

We collected information on sociodemographic, clinical and exposure data for each case from medical records, shift schedules, and case interviews, including vaccination status (number of doses and dates of vaccination). Proportions were calculated for case characteristics, and age was reported with median and interquartile range (IQR).

Case and contact person pairs were identified through case interviews, review of bed occupancy and shift schedules. For each new case, we identified all contacts during the infectious period. Additionally, we retrospectively assessed potential exposures to confirmed cases. For contact persons exposed to multiple cases, exposures involving prolonged interactions, absence of masks, or the initial case in the cluster were used to determine inclusion in the transmission network. The epicontacts package in the R software (version 4.1.3) was used to visualize transmission chains [26].

We calculated SARs based on the number of contact persons with a positive test result within 14 days after exposure to a case. Contact events identified during the infectious period of new cases were included in the analysis of SARs; retrospective assessments of potential exposures to confirmed cases were not considered.

We only present SARs for HA, ensuring the reliability of our findings while acknowledging the limitations encountered in the investigation of the much larger and rapidly evolving outbreak in HB.

### 2.6. Ethics

The outbreak investigations were conducted under the official mandate of the local PHA resulting from paragraph 25 section 1 sentence 1 German Infection Protection Act (IfSG) and were thus exempt from ethics committee review. Support by the Robert Koch Institute was provided after official request. We followed local and RKI ethics and data protection standards.

## 3. Results

### 3.1. Infection Prevention and Control Measures

In both hospitals, frequent meetings of the outbreak team and close coordination between the local PHA and the hospital took place, with case-by-case decisions being made and orders issued by the local PHA. FFP2 masks were mandatory.

#### 3.1.1. Hospital A

Three cases were identified on 18 January 2021 in HA’s cardiology ward. Following additional cases, the ward was quarantined on 21 January 2021. By 22 January 2021, 13 cases had been identified, and admissions to the hospital were stopped. To prevent any contact between employees, including agency staff, and the general population, a shuttle service between the hospital and their homes was provided (“shuttle quarantine”). On 22 January 2021, biweekly PCR screening of all the staff and patients began, shifting to weekly from 3 February 2021. The agency staff were contacted through their agencies and tested at their workplaces. The contacts were quarantined for 16 days, with nasopharyngeal swabs taken between days 10 and 16 when possible. The facilities to which discharged patients were transferred from 1 January 2021 onwards were informed about the outbreak. All the discharged patients from HA were to be quarantined as contact persons. The patients undergoing dialysis, chemotherapy, or other necessary readmissions at HA were placed in shuttle quarantine and tested during each hospital visit using rapid antigen tests and PCR.

#### 3.1.2. Hospital B

All the patients admitted to HB from HA after 1 January 2021 were identified, tested and isolated. Following the occurrence of cases in the pneumology and gastroenterology wards, both wards were closed, and the patients were quarantined on 28 January 2021 and 3 February 2021, respectively, with the patients screened for the virus twice a week. On 4 February 2021, following additional cases on the neurology, COVID-19, and hemato-oncology wards, the internal medicine unit, including the emergency department’s internal medicine section, stopped admissions. This was followed by the closure of the entire emergency department on 12 February 2021. Patients on the psychiatric ward and outpatients undergoing chemotherapy and immunosuppressive treatment continued to be admitted. From 6 February 2021, staff were only assigned to dedicated areas. A shuttle quarantine was provided for employees who had contact with cases from 23 January 2021 to 26 January 2021. Contact patients were discharged only after a 16-day quarantine and a negative PCR to prevent the spread of the virus outside the hospital. All the employees, including cleaning, service, and agency staff, underwent daily rapid antigen testing. From 6 February 2021, all the employees and, from 10 February 2021, all the patients were PCR-tested twice weekly. The agency staff were reached through their agencies and screened accordingly. On 22 February 2021, admission was stopped in the psychiatric ward.

### 3.2. Descriptive Epidemiology

**Figure 1:**
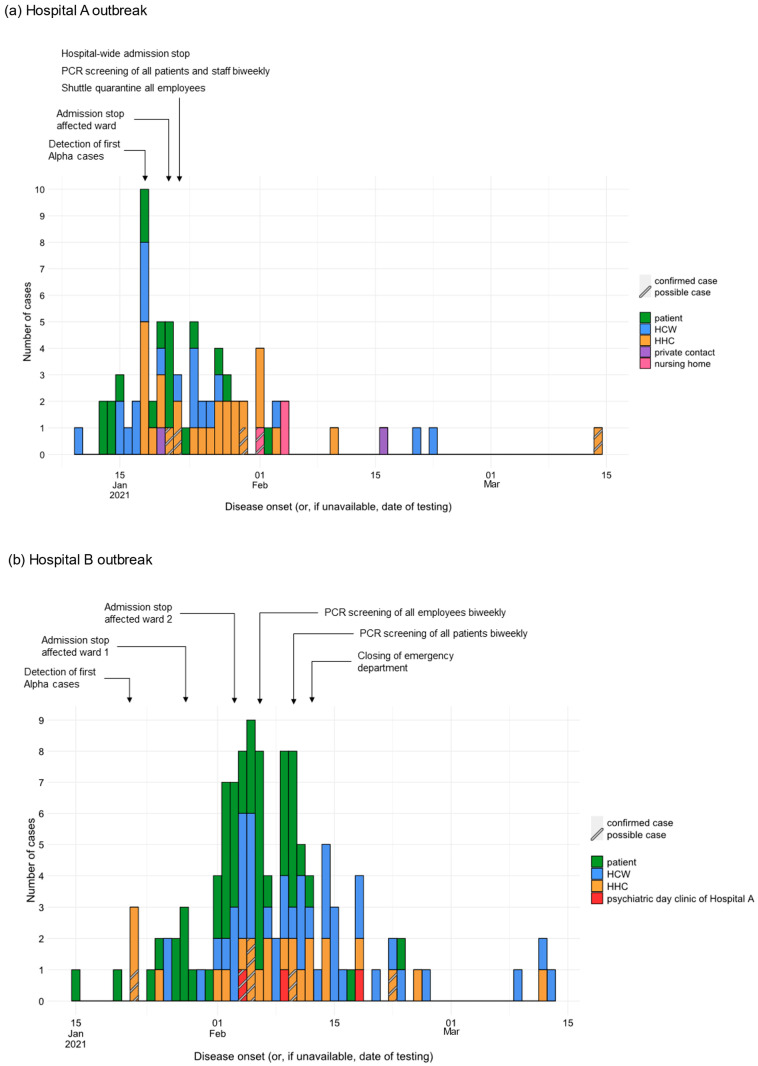
Epidemic curve for confirmed and possible SARS-CoV-2 outbreak cases by case category and event timeline for implemented measures. (**a**) Hospital A (*n* = 71). (**b**) Hospital B (*n* = 118). Germany January–March, 2021. HHC: household contact; HCW: healthcare worker.

By 13 March 2021, 66 confirmed and five possible cases related to HA were identified: 18 patients (25%), 20 HCWs (28%), 28 HHCs (39%) and two private contacts other than HHCs (3%) (Figure 1). Of all the patient cases, 16 were associated with the cardiology ward, one with the admission ward, and one with a non-cardiology ward and the intensive care unit (ICU). The outbreak spread to a nursing home via an HHC working there, resulting in two additional cases among the residents (4%). Related to HB, 112 confirmed and six possible cases were identified by 15 March 2021: 48 patients (41%), 43 HCWs (36%), and 24 HHCs (20%) (Figure 1). The outbreak spread to the psychiatric day clinic of HA via an HHC working there, where three cases (3%) were confirmed. Within the patient cases, the main affected wards were gastroenterology (*n* = 19), pneumology (*n* = 17), and neurology (*n* = 6). Further cases among the patients and HCWs occurred in the ICU, COVID-19 ward, surgical ward, and the hematology–oncology ward. Most HCW cases worked on the COVID-19 ward (*n* = 13). Cases (*n* = 9) resulting from patient–household member contacts occurred only from the primary case and from patient cases with discharge prior to the implementation of control measures for patient discharge on 6 February 2021.

Table 1 summarizes the case characteristics. In HA, the median age was 38 years (IQR = 26–69) for outbreak cases and 80 years (IQR = 73–86) for deceased cases. Screening identified six pre-symptomatic HCWs and patients, four of whom were detected after hospital-wide screening. Three asymptomatic cases were found, one after hospital-wide screening. In HB, the outbreak cases had a median age of 54 years (IQR = 36–78) and deceased cases 78 years (IQR = 71–82). Screening detected 18 pre-symptomatic cases; three were identified post-hospital-wide screening. Additionally, 13 asymptomatic cases were found, five following hospital-wide screening.

At the time, only one HCW per hospital had received two doses of COVID-19 vaccine.

### 3.3. Phylogenetic Analysis

**Figure 2:**
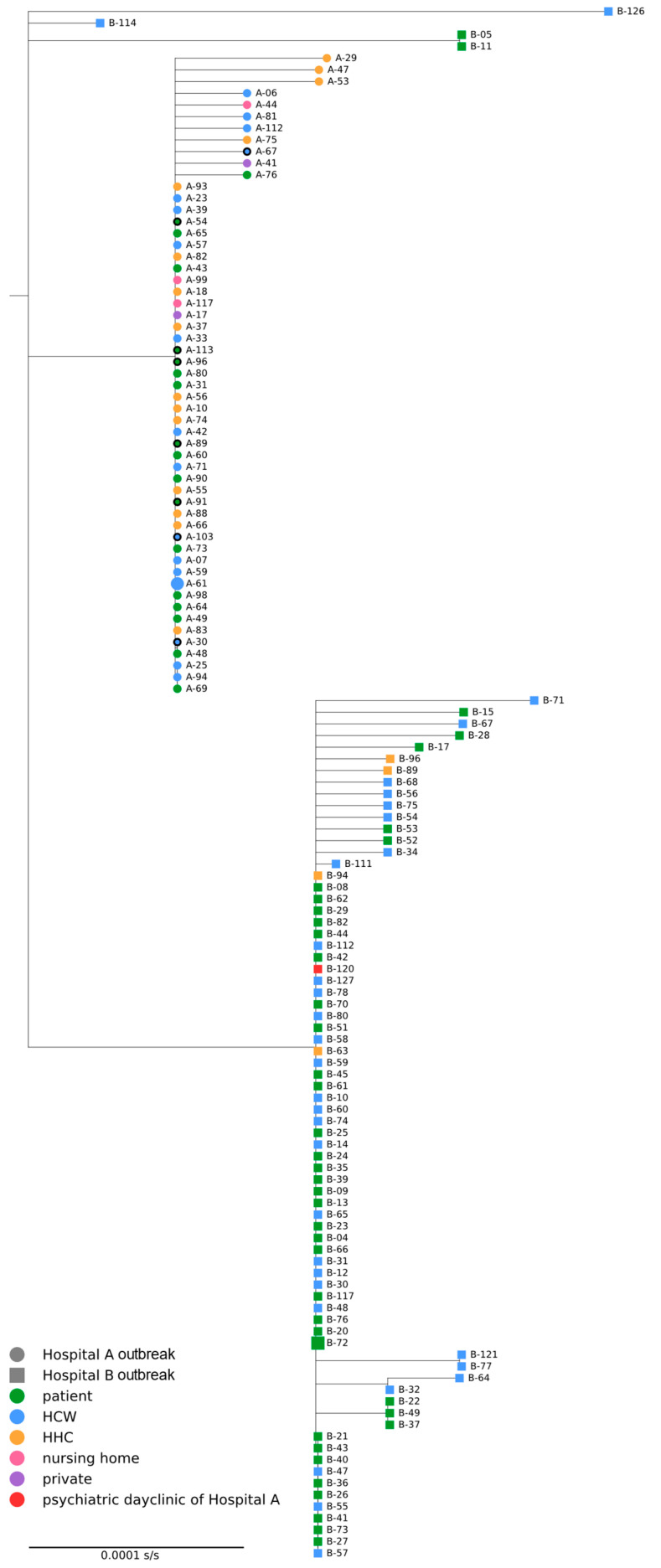
Maximum likelihood tree of sequences associated with the SARS-CoV-2 outbreaks in Hospitals A and B, Germany, January–March, 2021. Sequences associated with Hospital A are shown as circles, sequences from the outbreak in Hospital B as squares. Symbols are colored according to the case category. Suspected primary cases for the outbreak in Hospital A and the large cluster in Hospital B are indicated by larger symbols. A black outline indicates symbols of patients possibly infected by patient A-91. Nodes with IQ-TREE2 ultrafast bootstrap support <95% and SH-aLRT (SH-approximate likelihood-ratio test) values < 80% are collapsed. The tree is rooted with Wuhan-Hu1 (GISAID accession no. EPI_ISL_402125). HCW: healthcare worker; HHC: household contact.

We generated 55 SARS-CoV-2 consensus genomes with >90% genome coverage from samples associated with HA (83% of the confirmed cases in HA, 55/66) and 78 for samples associated with HB (70% of the confirmed cases in HB, 78/112). Figure 2 shows the resulting phylogenetic tree. The sequences associated with HA were monophyletic. For the sequences associated with HB, the tree shows one large clade of 74 sequences, one clade of two sequences and two singletons, indicating at least four potential and independent introductions into HB. The outbreak at HA was not linked to the outbreak at HB.

### 3.4. Transmission Network and Secondary Attack Rates

**Figure 3:**
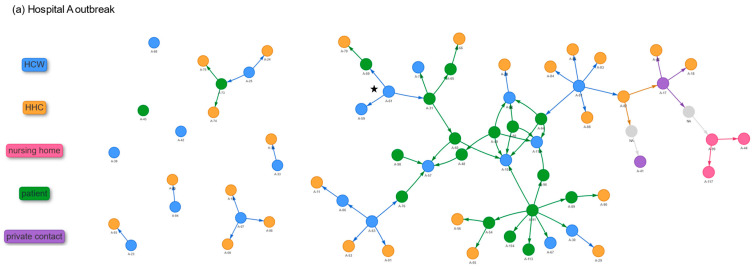

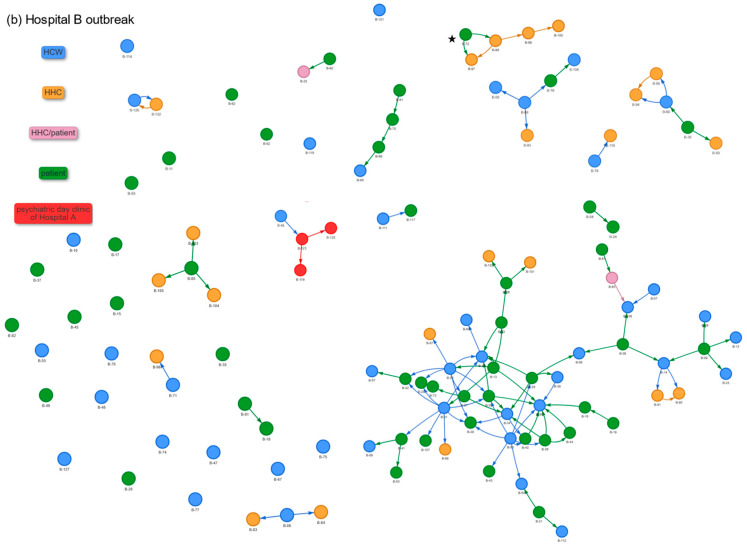
Transmission network of SARS-CoV-2 outbreak cases in (**a**) Hospital A and (**b**) Hospital B, Germany, January–March 2021. Each node represents an infected case, and the arrows indicate the likely direction of transmission between cases. Nodes with unknown identity (NA) are shown in grey. HCW: healthcare worker; HHC: household contact. The presumed primary case is marked with a star (the primary case is assumed based on contact tracing and onset of disease).

In the HA outbreak, contact tracing identified exposures for 54 cases, while 17 (11 HCWs and six patients) had no traceable source (Figure 3). Patient A-91 likely caused multiple infections, spreading to five patients (A-54, A-89, A-96, A-104, and A-113) and three HCWs (A-30, A-67, and A-103). A-91 was in a four-bed room on the cardiology ward, where two exposed patients changed rooms. Later moved to the ICU, A-91 likely infected two HCWs (A-30 and A-67). In HB, exposures were identified for 73 cases, while 45 remained unlinked (20 HCWs and 25 patients). Contact tracing indicates that an agency staff member (B-111) working on the COVID-19 and later psychiatric wards may have spread the outbreak from the somatic to psychiatry wards. In both hospitals, transmission occurred among patients, HCWs, and between them. Patient-to-HCW transmission is suspected in seven cases in the HA outbreak and in 18 cases in the HB outbreak.

SARs for HA outbreak are shown in Table 2.

### 3.5. Potential Sources of the Outbreaks

Based on the available epidemiological data, there are two hypotheses for the introduction of the SARS-CoV-2 Alpha variant in HA: (1) an employee (A-61) on the admission ward with the earliest symptom onset (10 January 2021) among the known cases, or (2) a SARS-CoV-2-positive patient whose sample was not available for confirmatory testing but from whom a contact person was attributed to the outbreak (A-98) (Figure 2). Phylogenetic analysis indicates that sequence A-61 likely represents an early case in the outbreak.

In the HB outbreak, patient B-05—transferred from a hospital abroad—was initially considered as the primary case, but phylogenetics excluded them from the main cluster. Sequencing also showed no transmission from HA transfers (A-89, A-90, and A-69). The larger cluster in HB originated from patient B-72, who required resuscitation upon admission (Figure 2). Although B-72 tested positive on admission, Alpha variant infection was confirmed only 14 days later. The initial testing showed only one of two mutations (S:N501Y-negative), leading to exclusion as the primary case. Sequencing later confirmed Alpha. Resuscitation took place in a COVID-19 ward, where protective measures were assumed to be in place but multiple transmissions likely occurred.

## 4. Discussion

In January 2021, Germany’s first reported nosocomial SARS-CoV-2 Alpha variant outbreak occurred in HA. As Alpha was not yet established in the population, extensive measures were taken to contain its spread within the hospital and prevent community transmission. A second outbreak in the nearby HB was reported only shortly afterwards, characterized by different dynamics. The outbreak in HB was more extensive, primarily impacting patients and staff, while the outbreak in HA was smaller in scale, mainly involving household cases. Both outbreaks were effectively contained within a relatively short period—approximately one month in HA and two months in HB—despite the early emergence of the Alpha variant and the absence of widespread vaccination at the time.

The outbreak at HB likely spread extensively within the hospital as the primary case required resuscitation shortly after admission. The patient first tested positive for SARS-CoV-2, with Alpha confirmed later. Intensified infection control measures focused on Alpha cases, and the resuscitation took place in the COVID-19 ward, where it was assumed that protective measures were followed. This underscores the need for prompt contact tracing and swift action in dynamic situations, especially when compliance with infection control standards may be compromised. Other investigations support this by showing that, in the nosocomial setting, a limited number of individuals and special events can contribute to a high number of transmissions [27,28] and that optimizing contact tracing can reduce nosocomial transmission [29]. Detailed documentation of cases and contacts and implemented interventions should be maintained to allow continuous evaluation and inform future outbreak management.

The wider spread in HB may also reflect the gradual implementation of control measures, in contrast to HA, where rigorous hospital-wide action was promptly enacted. This emphasizes the need for an early and decisive response in nosocomial outbreaks, as previously described [10,30]. However, it is crucial to recognize that a hospital-wide admission stop can harm the community by delaying medical treatment, straining other healthcare facilities, and potentially worsening patient outcomes due to the lack of access to timely medical care [31].

Although the two outbreak peaks were only 2.5 weeks apart, timing mattered: during the HB outbreak, Alpha was already widespread, and containment efforts had slowed, making rigorous action difficult—especially due to the community impact of a hospital closure.

In both outbreaks, screening effectively identified pre- and asymptomatic individuals, potentially averting further transmission. This highlights the need for rigorous testing protocols during outbreaks, supported by previous research on the effectiveness of screening in preventing nosocomial transmission [32,33]. Clear criteria for testing frequency during outbreaks should be established to ensure timely detection of cases.

The case search among agency personnel proved to be difficult, mainly due to communication challenges with different agencies. Additionally, ensuring that agency staff worked only in designated areas was difficult to oversee. As seen in HB, transferring an agency staff HCW from the COVID-19 ward to the psychiatric unit may have contributed to cases in psychiatry. These operational challenges are consistent with findings from other settings, where the employment of agency staff was associated with significantly higher SARS-CoV-2 infection rates and increased odds of outbreaks [34]. A clear communication strategy and designated responsibilities are crucial to ensure rapid decision-making, coordination, and better oversight of agency staff assignments.

Especially during the first wave of the pandemic, front-line HCWs were at increased risk of COVID-19 [35,36]. Prolonged and close contact with infected persons, handling of secretions, as well as high-risk procedures can increase the risk of respiratory infections in HCWs [37]. By the time COVID-19 patients are admitted to COVID-19 wards, their infection is known and staff are specially trained and prepared. However, 13 HCW cases were found at HB on the COVID-19 ward, underscoring the need for a safer work environment—especially during high workloads, increased COVID-19 admissions, and high-risk procedures like resuscitation.

Transmissions of SARS-CoV-2 to other patients are largely driven by patients who acquired a SARS-CoV-2 infection in the hospital [1,38,39]. We observed high SARs among patient-to-patient contacts, also described by other studies [39]. Additionally, we observed a notable number of transmissions from patients to HCWs. This suggests that patients who acquire the infection in the hospital pose a considerable transmission risk, likely due to higher viral loads in newly infected individuals and prolonged contact times. Our findings also indicate cases linked to shared rooms and intra-ward transfers, resulting in multiple patient-to-patient contacts. Guidelines for urgent patient transfers should be established to minimize the risk of cross-ward transmission. During high-incidence periods, it is crucial to reduce transmission risk by limiting room transfers and ensuring early case detection through routine testing and symptom monitoring in shared rooms. High SARs among HHCs were observed in the HA outbreak. In the HB outbreak, household transmission was reduced through prolonged isolation and discharge after a negative PCR—measures that can help to limit community spread if resources permit.

This study has several limitations. First, not all the cases were successfully sequenced, and therefore phylogenetic resolution is incomplete. Nevertheless, the available sequence and epidemiological data clarified transmissions and excluded the suspected primary case of the HB outbreak from the main cluster. Second, the epidemiological data were obtained through interviews and record review and are therefore subject to recall and documentation bias, but these data provided detailed insights into case characteristics and exposure patterns. Third, despite intensive contact tracing, some contacts may have been missed. Fourth, we did not apply statistical transmission models; instead, we present a descriptive reconstruction of likely transmission events. The introduction of Alpha in HA by unidentified cases cannot be ruled out as early infections may have gone undetected before Alpha testing began. Finally, we did not perform a quantitative assessment of the effectiveness of specific IPC measures (e.g., ward closures). Interventions were implemented as bundles during an emergency response. Nonetheless, our observations provide practical guidance on effective outbreak control strategies.

## 5. Conclusions

To conclude, our results provide valuable data-driven lessons for control measures in nosocomial viral respiratory outbreaks. First, they highlight the relevance of consistent contact tracing, especially in high-risk emergency situations that can lead to superspreading events, such as the resuscitation of the primary case in the HB outbreak. Second, our results underline the importance of early and decisive actions to control nosocomial outbreaks. Third, our findings highlight the need for wide-scale testing during outbreaks, including asymptomatic individuals. Fourth, they emphasize the role of transmissions from patients with healthcare-acquired infections and demonstrate the importance of assuring a safe working environment for HCWs and minimizing patient movements and multi-bed patient rooms. Finally, these findings highlight the importance of effective communication with external agencies and improved oversight of agency staff assignments. Our investigations took place in the early stages of the pandemic before the roll-out of vaccinations, when the SARS-CoV-2 Alpha variant had just emerged and efforts were made to stop transmission of this new variant in the community. Since this early phase of the pandemic, widespread immunity to SARS-CoV-2 has been achieved, and mortality and morbidity from COVID-19 have significantly decreased [40,41]. However, the timing of our study, conducted before widespread population immunity, represents a key strength, providing valuable insights into controlling nosocomial outbreaks caused by emerging highly transmissible respiratory viruses and highlighting key considerations for hospital preparedness and response.

## Figures and Tables

**Table 1:** Characteristics of SARS-CoV-2 outbreak cases by case category, Hospital A and Hospital B, Germany, January–March, 2021.

Characteristic	Hospital A										Hospital B									
	Patient N = 18		HCW N = 20		HHC/Private Contacts N = 30		Nursing Home N = 3		Total N = 71		Patient N = 48		HCW N = 43		HHC N = 24		Psychiatric Day Clinic of Hospital A N = 3		Total N = 118	
	*n*	%	*n*	%	*n*	%	*n*	%	*n*	%	*n*	%	*n*	%	*n*	%	*n*	%	*n*	%
**Sex**																				
Male	9	50%	6	30%	17	57%	0	0%	32	45%	29	60%	10	23%	11	46%	1	33%	51	43%
Female	9	50%	14	14%	13	43%	3	100%	39	55%	19	40%	33	77%	13	54%	2	67%	67	57%
**Symptomatic**																				
Yes	15	83%	19	95%	26	87%	3	100%	63	89%	38	79%	36	84%	23	96%	3	100%	100	85%
No	3	17%	0	0%	3	10%	0	0%	6	8%	8	17%	5	12%	1	4%	0	100%	14	12%
Missing	-	-	1	5%	1	3%	-	-	2	3%	2	4%	2	5%	-	-	-	-	4	3%
**Intensive care treatment**																				
Yes	2	11%	0	0%	3	10%	0	0%	5	7%	10	21%	1	2%	1	4%	0	0%	12	10%
No	15	83%	20	100%	27	90%	3	100%	65	92%	37	77%	42	98%	23	96%	3	100%	105	89%
Missing	1	6%	-	-	-	-	-	-	1	1%	1	2%	-	-	-	-	-	-	1	1%
**Deceased**																				
Yes	8	44%	0	0%	2	7%	2	67%	12	17%	19	40%	0	0%	1	4%	0	0%	20	17%
No	10	56%	20	100%	28	93%	1	33%	59	83%	29	60%	43	100%	23	96%	3	100%	98	83%

HCW: healthcare worker; HHC: household contact.

**Table 2:** Secondary attack rates, SARS-CoV-2 outbreak Hospital A, Germany, January–March 2021.

Exposure	Number of Contacts	Number of Cases Originating from These Contacts	Secondary Attack Rate (%)
**Hospital**	96	13	14%
HCW-to-HCW	10	1	10%
HCW-to-patient	28	3	11%
Patient-to-HCW	33	1	3%
Patient-to-patient	25	8	32%
**Nursing home**	36	2	6%
**Household**	32	24	75%
**Private (other than household)**	4	1	25%

HCW: healthcare worker.

## Data Availability

Sequences generated as part of this study are available on GISAID. The following pairs indicate each case and its corresponding GISAID accession number (Case—Accession Number): A-06—EPI_ISL_5947119; A-07—EPI_ISL_5947235; A-10—EPI_ISL_19813456; A-103—EPI_ISL_19813470; A-112—EPI_ISL_5947088; A-113—EPI_ISL_912262; A-117—EPI_ISL_19813465; A-17—EPI_ISL_19813474; A-18—EPI_ISL_19813464; A-23—EPI_ISL_5947177; A-25—EPI_ISL_5947234; A-29—EPI_ISL_19813423; A-30—EPI_ISL_5947114; A-31—EPI_ISL_5947157; A-33—EPI_ISL_5947232; A-37—EPI_ISL_19813475; A-39—EPI_ISL_1989651; A-41—EPI_ISL_1989413; A-42—EPI_ISL_912263; A-43—EPI_ISL_5947102; A-44—EPI_ISL_19813466; A-47—EPI_ISL_19813472; A-48—EPI_ISL_5947105; A-49—EPI_ISL_5947233; A-53—EPI_ISL_19813458; A-54—EPI_ISL_1840664; A-55—EPI_ISL_19813426; A-56—EPI_ISL_19813471; A-57—EPI_ISL_1989432; A-59—EPI_ISL_5947156; A-60—EPI_ISL_5947093; A-61—EPI_ISL_19813457; A-64—EPI_ISL_5947091; A-65—EPI_ISL_19813425; A-66—EPI_ISL_19813424; A-67—EPI_ISL_19813467; A-69—EPI_ISL_5947242; A-71—EPI_ISL_912260; A-73—EPI_ISL_5947084; A-74—EPI_ISL_19813450; A-75—EPI_ISL_19813468; A-76—EPI_ISL_5947180; A-80—EPI_ISL_5947094; A-81—EPI_ISL_5947237; A-82—EPI_ISL_19813476; A-83—EPI_ISL_19813459; A-88—EPI_ISL_19813469; A-89—EPI_ISL_5947083; A-90—EPI_ISL_5947241; A-91—EPI_ISL_5947147; A-93—EPI_ISL_19810916; A-94—EPI_ISL_5947236; A-96—EPI_ISL_5947082; A-98—EPI_ISL_5947087; A-99—EPI_ISL_19813460; B-04—EPI_ISL_5947127; B-05—EPI_ISL_5947085; B-08—EPI_ISL_5947244; B-09—EPI_ISL_5947153; B-10—EPI_ISL_5947243; B-11—EPI_ISL_5947167; B-111—EPI_ISL_1989406; B-112—EPI_ISL_1989270; B-114—EPI_ISL_1989271; B-117—EPI_ISL_1989338; B-12—EPI_ISL_5947168; B-120—EPI_ISL_1989389; B-121—EPI_ISL_5947246; B-126—EPI_ISL_1986421; B-127—EPI_ISL_1986434; B-13—EPI_ISL_1885941; B-14—EPI_ISL_19760522; B-15—EPI_ISL_5947245; B-17—EPI_ISL_5947248; B-20—EPI_ISL_5947181; B-21—EPI_ISL_5947250; B-22—EPI_ISL_5947249; B-23—EPI_ISL_5947173; B-24—EPI_ISL_5947252; B-25—EPI_ISL_5947251; B-26—EPI_ISL_5947253; B-27—EPI_ISL_5947259; B-28—EPI_ISL_5947257; B-29—EPI_ISL_1989676; B-30—EPI_ISL_5947194; B-31—EPI_ISL_5947193; B-32—EPI_ISL_1989516; B-34—EPI_ISL_5947258; B-35—EPI_ISL_5947255; B-36—EPI_ISL_5947256; B-37—EPI_ISL_5947260; B-39—EPI_ISL_5947265; B-40—EPI_ISL_1989677; B-41—EPI_ISL_5947261; B-42—EPI_ISL_5947263; B-43—EPI_ISL_5947264; B-44—EPI_ISL_5947262; B-45—EPI_ISL_1989666; B-47—EPI_ISL_5947254; B-48—EPI_ISL_1989669; B-49—EPI_ISL_1989543; B-51—EPI_ISL_1989513; B-52—EPI_ISL_1989520; B-53—EPI_ISL_1989561; B-54—EPI_ISL_1989563; B-55—EPI_ISL_1989670; B-56—EPI_ISL_1989527; B-57—EPI_ISL_5947266; B-58—EPI_ISL_1989509; B-59—EPI_ISL_1989538; B-60—EPI_ISL_1989532; B-61—EPI_ISL_1989424; B-62—EPI_ISL_1989506; B-63—EPI_ISL_1989541; B-64—EPI_ISL_1989411; B-65—EPI_ISL_1989526; B-66—EPI_ISL_1989537; B-67—EPI_ISL_1989511; B-68—EPI_ISL_1989554; B-70—EPI_ISL_1989430; B-71—EPI_ISL_1989540; B-72—EPI_ISL_5947086; B-73—EPI_ISL_5947267; B-74—EPI_ISL_1989546; B-75—EPI_ISL_1989452; B-76—EPI_ISL_1989450; B-77—EPI_ISL_1989549; B-78—EPI_ISL_1989404; B-80—EPI_ISL_1989438; B-82—EPI_ISL_1986302; B-89—EPI_ISL_1353110; B-94—EPI_ISL_1353121; B-96—EPI_ISL_1353046.
